# Supplementary material for: Estradiol Modulates the Sensitivity to Vancomycin of Lactobacillus paracasei and Staphylococcus aureus Biofilms—Constituents of Human Skin and Vaginal Microbiota
Source: Microorganisms. 2025 Dec 5;13(12):2777. doi: 10.3390/microorganisms13122777 (PMC12736244; doi:10.3390/microorganisms13122777)
Supplement: Supplementary file 1 [file microorganisms-13-02777-s001.zip › Table S1.pdf]

Table S1. Description of selected antibiotic resistance genes and primers used for qPCR of *S. aureus*.

Gene, antibiotic, mechanism	Primer	Sequence 5’-3’
mepR (tetracyclines) – antibiotic efflux	Forvard	GCATTACAACGAACAGGTCCA
	Reverse	TCCCAGAGGTAGTCAGCCC
mgrA (peptide antibiotics, tetracyclines,	Forvard	AGCGTGAACGTTCCGAAGTC
cephalosporines) – antibiotic efflux	Reverse	GAAGCTGAAGCGACTTTGTCA
norC (fluoroquinolones) – antibiotic efflux	Forvard	TTGTTGTTGGAGCAGGTGGT
	Reverse	CAGGCGTCCCTTTGATGAGT
vanTG (glycopeptides) – antibiotic target alteration	Forvard	CTTTGCCTGTGCTGACGAAC
	Reverse	ACCTCTACCGACTGTGGACT
lmrS (oxazolidine, macrolides, phenicols,	Forvard	TGGACCTGCGCTGCTTATAC
aminoglycosides) – antibiotic efflux	Reverse	AGCCGTGCCATGTGAGATTT
sdrM (fluoroquinolones) – antibiotic efflux	Forvard	TGGGCATAGTTGGCAGTGTT
	Reverse	ATGGCAATGATCGCAATCGG
16 s rRNA	Forvard	TCAACCGTGGAGGGTCATTG
	Reverse	TGCACCACCTGTCACTTTGT
