# Supplementary material for: Estradiol Modulates the Sensitivity to Vancomycin of Lactobacillus paracasei and Staphylococcus aureus Biofilms—Constituents of Human Skin and Vaginal Microbiota
Source: Microorganisms. 2025 Dec 5;13(12):2777. doi: 10.3390/microorganisms13122777 (PMC12736244; doi:10.3390/microorganisms13122777)
Supplement: Supplementary file 1 [file microorganisms-13-02777-s001.zip › Supplementary data S1.pdf]

Supplementary Data S1. The protocol of biofilm matrix isolation.

After incubation, biofilms were collected into 50 mL conical tubes (Corning, New York, USA) and resuspended in sterile Milli-Q water to a final volume of 10 mL. Samples were sonicated using a SoniPrep 150 Plus ultrasonic disintegrator (MSE, London, UK) with a titanium probe for 1 min at an amplitude of 7.8 µm. An ice bath was used to prevent overheating. Sonicated samples were transferred to polypropylene ultracentrifuge tubes (Beckman, Brea, CA, USA) and layered onto a CsCl gradient prepared as described previously [38]. Four CsCl solutions (46%, 28%, 14%, and 7%) were layered, and the sample was applied on top. Ultracentrifugation was performed in an Avanti-J30i (Beckman) at 100000 × *g* and 22 °C for 1 h. The supernatant was transferred to new tubes and residual cells were removed by centrifugation at 3800 × *g* for 20 min at room temperature.

To assess the completeness of matrix isolation, an LDH assay (Vital, Russia) was performed as described [42]. Cell suspensions were adjusted to match the OD_{340} of the isolated matrix, and assay results were compared in a 96-well plate using the kit protocol.

References

38. Gannesen, A.V.; Ziganshin, R.H.; Zdorovenko, E.L.; Klimko, A.I.; Ianutsevich, E.A.; Danilova, O.A.; Tereshina, V.M.; Gorbachevskii, M.V.; Ovcharova, M.A.; Nevolina, E.D.; et al. Epinephrine extensively changes the biofilm matrix composition in *Micrococcus luteus* C01 isolated from human skin. *Front. Microbiol.* **2022**, *13*, 1003942. https://doi.org/10.3389/fmicb.2022.1003942.

42. Gannesen, A.V.; Zdorovenko, E.L.; Botchkova, E.A.; Hardouin, J.; Massier, S.; Kopitsyn, D.S.; Gorbachevskii, M.V.; Kadykova, A.A.; Shashkov, A.S.; Zhurina, M.V.; et al. Composition of the biofilm matrix of *Cutibacterium acnes* acneic strain RT5. *Front. Microbiol.* **2019**, *10*, 1284. https://doi.org/10.3389/fmicb.2019.01284.
