# Supplementary material for: Estradiol Modulates the Sensitivity to Vancomycin of Lactobacillus paracasei and Staphylococcus aureus Biofilms—Constituents of Human Skin and Vaginal Microbiota
Source: Microorganisms. 2025 Dec 5;13(12):2777. doi: 10.3390/microorganisms13122777 (PMC12736244; doi:10.3390/microorganisms13122777)
Supplement: Supplementary file 1 [file microorganisms-13-02777-s001.zip › Supplementary data S2.pdf]

Supplementary data S2. The protocol of lipids analysis

The separation of polar lipids was conducted using two-dimensional thin-layer chromatography on silica gel 60 glass plates (Merck) in the following systems: chloroform–methanol–water (65:25:4) in the first direction and chloroform–acetone–methanol–acetic acid–water (50:20:10:10:5) in the second direction [44]. A total of 200-250 μg of lipids was applied to a plate. Neutral lipids were analyzed using one-dimensional thin-layer ascending chromatography on 10×10 cm silica gel 60 glass plates (Merck, Germany). For lipid separation, a hexane–diethyl ether–acetic acid (77:23:1) solvent system was used. A total of 125 μg of lipids was applied to a glass plate.

Chromatograms were visualized by spraying with 5% sulfuric acid in ethanol followed by heating at 180 °C for 15 min (total polar lipids). Lipids were identified using standards and diagnostic sprays: molybdenum blue for phospholipids (Dittmar-Lester modification [45]), ninhydrin for amino-containing lipids, alpha-naphthol for glycolipids, and Dragendorff's reagent for choline [46]. All chemicals were from Merck (Darmstadt, Germany).

To determine the general composition of polar and neutral lipids, components were visualized by spraying the plates with 5% H2SO4 (Rushim, Moscow, Russia) in ethanol (v/v) followed by heating for 15 min at 180 °C. For the identification of polar lipids, individual standard compounds were used and reactions with molybdene blue dye were conducted according to the modified Dittmer and Lester method [45] for phospholipids. Additionally, a reaction with ninhydrin was performed to identify aminolipids, an α-naphthol test was used for glycolipids, and Dragendorff's reagent was used to detect choline [46]. The individual phospholipid standards used were phosphatidylethanolamines, phosphatidylcholines, phosphatidylglycerols, and diphosphatidylglycerols (Larodan, Monroe, MI, USA). Neutral lipids were identified using individual markers for sterols; mono-, di-, and triacylglycerols; and free fatty acids (Sigma-Aldrich, Burlington, MA, USA).

Neutral lipids were analyzed by one-dimensional ascending TLC on Silica gel 60 glass plates (10 × 10 cm; Merck) using hexane:diethyl ether:acetic acid (77:23:1). A total of 160 μg lipid was loaded per plate.

44. Kates, M. Techniques of Lipidology: Isolation, Analysis and Identification of Lipids; Laboratory Techniques in Biochemistry and Molecular Biology; Elsevier: Amsterdam, The Netherlands, 1972; Volume 3, 267. https://doi.org/10.1016/S0075-7535(08)70544-8.

45. Benning, C.; Huang, Z.H.; Gage, D.A. Accumulation of a novel glycolipid and a betaine lipid in cells of *Rhodobacter sphaeroides* grown under phosphate limitation. *Arch. Biochem. Biophys.* **1995**, *317*, 103–111. https://doi.org/10.1006/abbi.1995.1141.

46. Vaskovsky, V.E.; Kostetsky, E.Y.; Vasendin, I.M. A universal reagent for phospholipid analysis. *J. Chromatogr*. **1975**, *114*, 129–141. https://doi.org/10.1016/s0021-9673(00)85249-8.
