# Supplementary material for: Estradiol Modulates the Sensitivity to Vancomycin of Lactobacillus paracasei and Staphylococcus aureus Biofilms—Constituents of Human Skin and Vaginal Microbiota
Source: Microorganisms. 2025 Dec 5;13(12):2777. doi: 10.3390/microorganisms13122777 (PMC12736244; doi:10.3390/microorganisms13122777)
Supplement: Supplementary file 1 [file microorganisms-13-02777-s001.zip › Supplementaty data S3.pdf]

Supplementaty data S3. Protocol for RNA isolation used in the study

Petri dishes containing RCM agar with or without active compounds were prepared. A sterile 21 mm diameter GMFF was placed on each plate. The suspension of *S. aureus* was prepared as described above, and 25 μL was inoculated onto the center of each filter. Biofilms were grown aerobically for 24 h at 33 °C. Total RNA was extracted using the Magen HiPure Total RNA Kit (Magen, Guangzhou, China) with modifications. After incubation, the filter with biomass was transferred to a porcelain mortar. RTL buffer with beta-mercaptoethanol (per manufacturer, Sigma, Burlington, MA, USA) was added, along with 0.5 cm^3 crushed glass. The mortar and pestle were cooled with liquid nitrogen. When approximately three quarters of the nitrogen had evaporated, the biomass was ground vigorously until the nitrogen was gone but before thawing. This freeze-grind cycle was repeated 4 to 5 times. One milliliter of RTL buffer was then added, mixed until thawed, and transferred to a 2 mL microcentrifuge tube (Eppendorf, Hamburg, Germany). Glass powder was pelleted in a MiniSpin (Eppendorf) at 11700 × g for 15 s at room temperature. The supernatant was applied to RNeasy columns and processed per the kit protocol. RNA quality was checked by agarose gel electrophoresis (1% agarose, 0.01% v/v ethidium bromide) in fresh 1× TAE buffer at 65 V for 70 min. rRNA bands were used as the primary quality marker and visualized on a Bio-Rad Gel Doc XR system with Quantity One 4.6.3. RNA was stored at −80 °C.
