# Supplementary figures and images for: Estradiol Modulates the Sensitivity to Vancomycin of Lactobacillus paracasei and Staphylococcus aureus Biofilms—Constituents of Human Skin and Vaginal Microbiota
Source: Microorganisms. 2025 Dec 5;13(12):2777. doi: 10.3390/microorganisms13122777 (PMC12736244; doi:10.3390/microorganisms13122777)

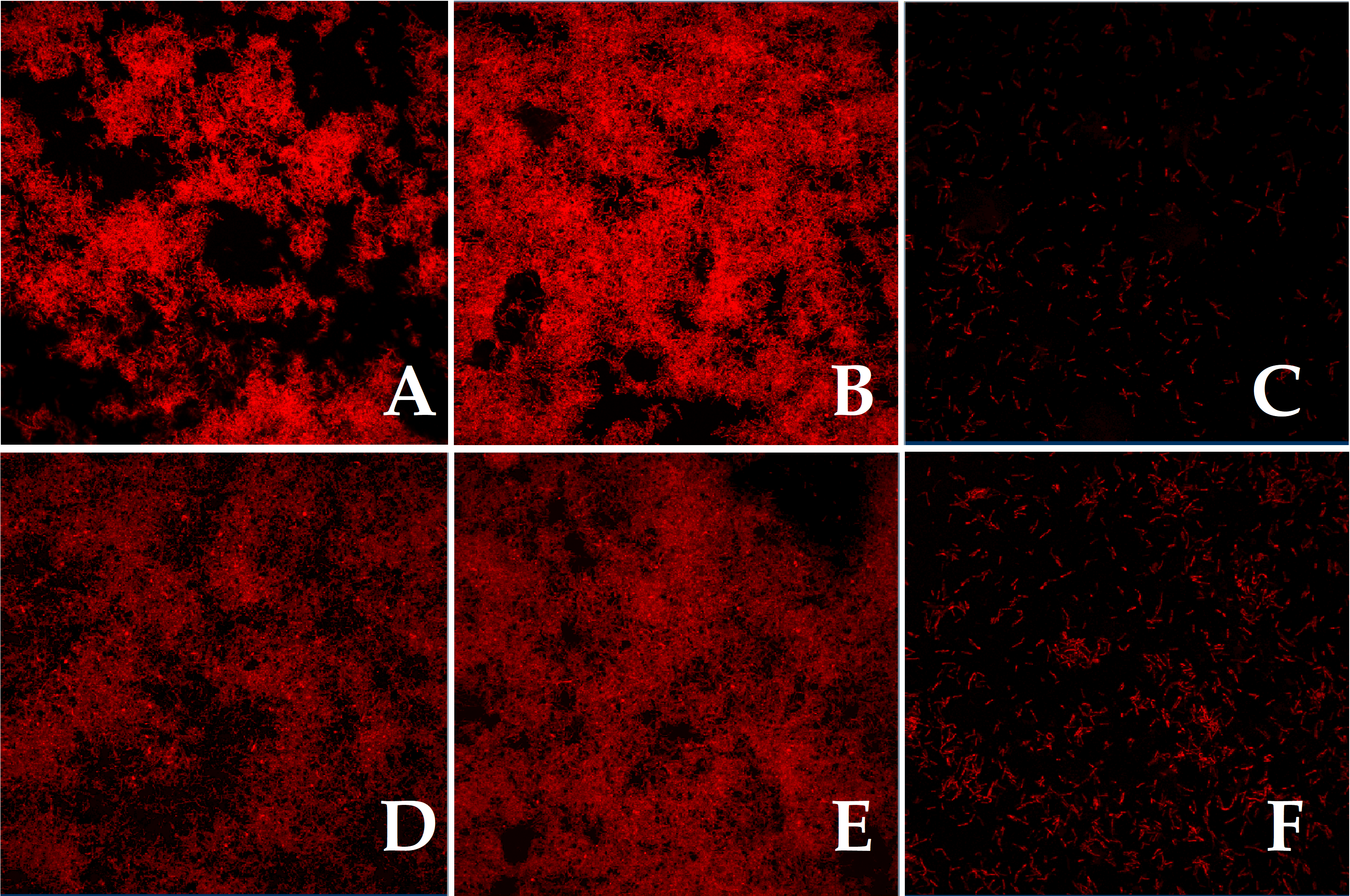

Supplement: Supplementary file 1 [file microorganisms-13-02777-s001.zip › Figure_S1.png]

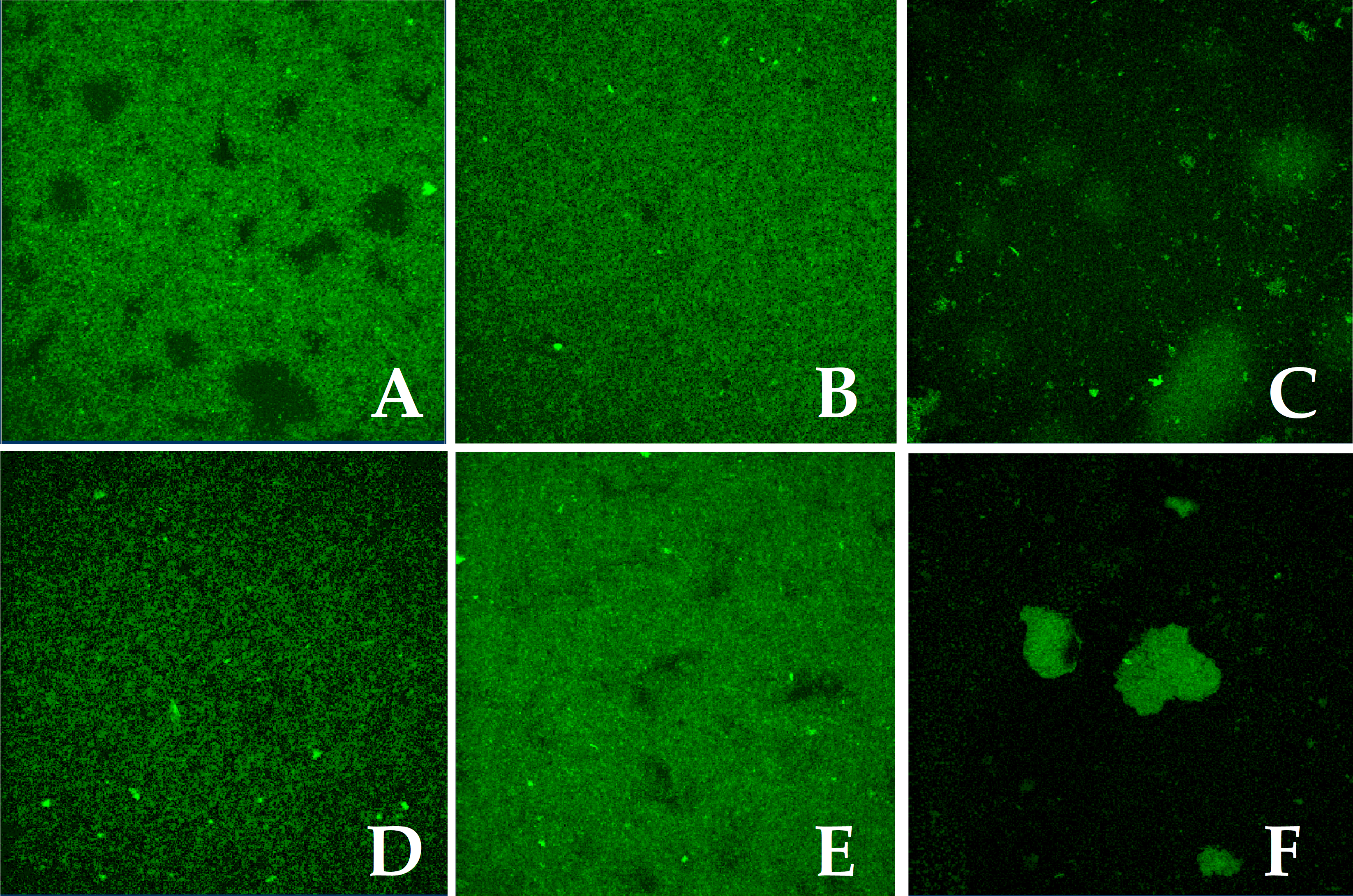

Supplement: Supplementary file 1 [file microorganisms-13-02777-s001.zip › Figure_S2.png]

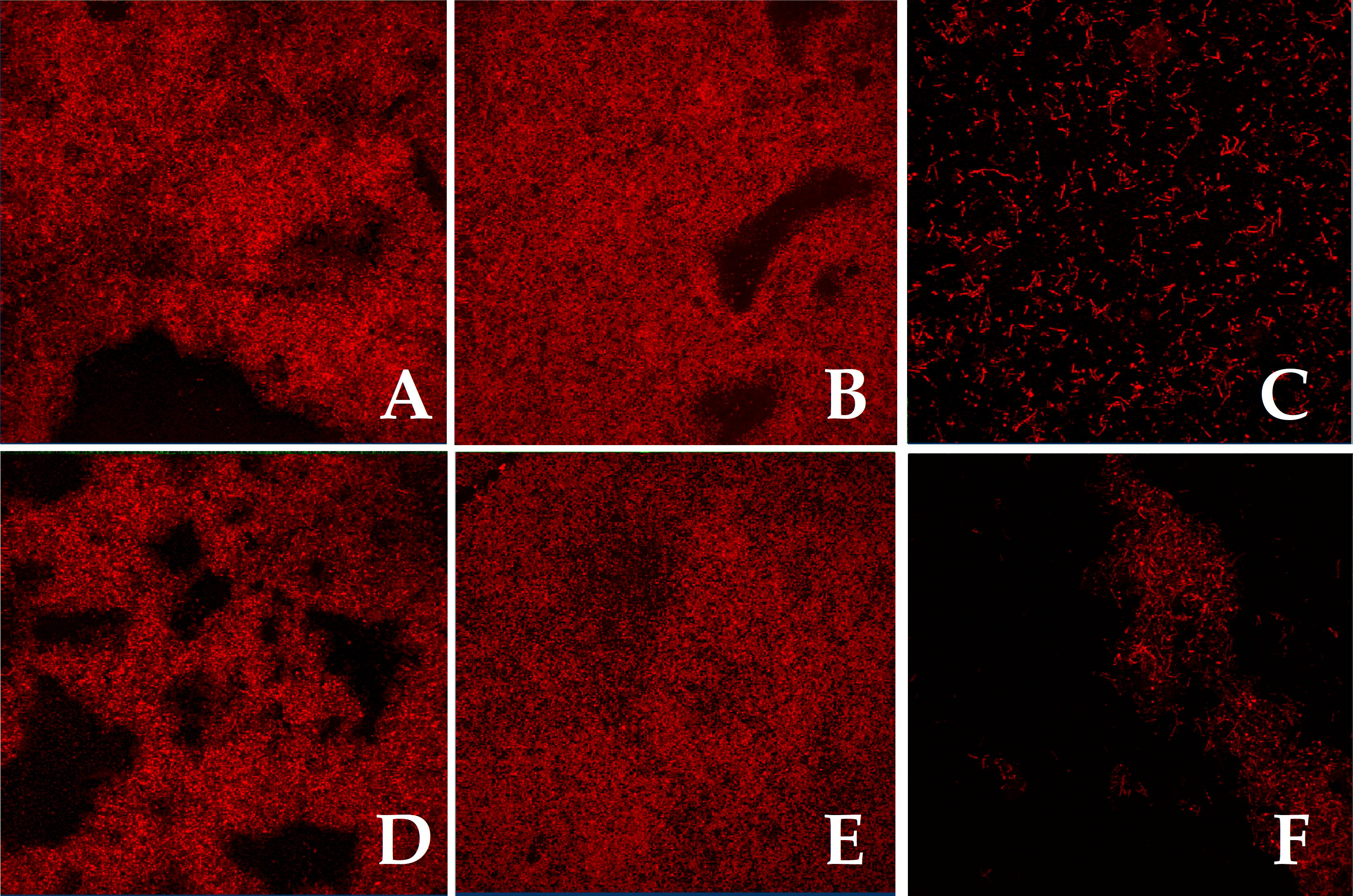

Supplement: Supplementary file 1 [file microorganisms-13-02777-s001.zip › Figure_S3.png]

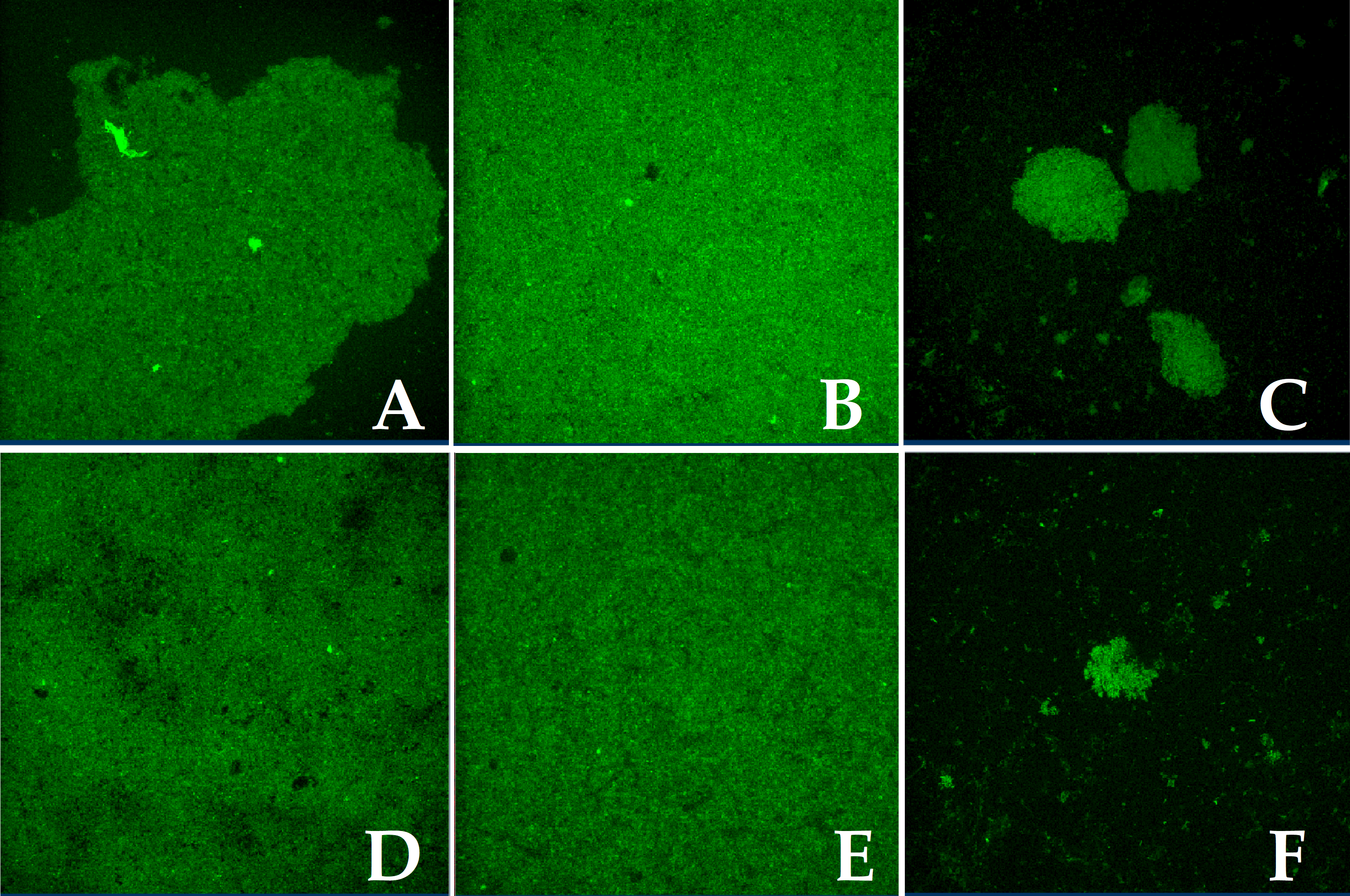

Supplement: Supplementary file 1 [file microorganisms-13-02777-s001.zip › Figure_S4.png]
